# Kinetics of rabies-neutralising antibodies in non-human primates after the injection of adjuvanted and inactivated rabies vaccine

**DOI:** 10.4102/ojvr.v93i1.2238

**Published:** 2026-02-16

**Authors:** Yacine Souissi, Mariem Handous, Mohamed Bayoudh, Sarra Torjemane, Médiha Khamassi Khbou

**Affiliations:** 1 Laboratory of Infectious Animal Diseases, Zoonoses and Sanitary Regulation, National School of Veterinary Medicine of Sidi Thabet, Manouba University, Ariana, Tunisia; 2 Rabies Laboratory, Pasteur Institute of Tunis, University of Tunis El Manar, Tunis, Tunisia; 3 Friguia Zoo Parc, Ennfidha, Sousse, Tunisia

**Keywords:** rabies, non-human primates, antibodies, vaccine, zoo

## Abstract

The main objective of this study is to evaluate the humoral response of 33 captive non-human primates from the Friguia zoo Park (Sousse district, Tunisia) following vaccination with an adjuvanted and inactivated rabies virus vaccine. The animals were sampled at day one, then at day 30, day 180 and day 365 post-vaccination. Antibody titration was performed using the FAVN (fluorescent antibody virus neutralisation) method in the national reference laboratory for rabies in Tunisia at the Pasteur Institute of Tunis. One-month post-vaccination, 100% of animals seroconverted, and 76.5% maintained a high titre up to 1 year. However, a decrease in titres was observed at day 180 and day 365, mainly in baboons and apes, respectively, whereas lemurs maintained a high titre up to 1-year post-vaccination.

**Contribution:**

In light of these results, we can recommend that the vaccination protocol for the primates at Friguia zoo Park be based on an initial vaccination with the adjuvanted and inactivated rabies virus vaccine, followed by an annual booster for lemurs and a semi-annual booster for baboons and apes.

## Introduction

Rabies is among the oldest human diseases in the world and still, until nowadays, one of the most fatal and feared disease (Baer 2007; Rupprecht et al. 2020). The rabies virus is a *Lyssavirus*, which causes symptoms associated to encephalomyelitis, leading to death in 100% of clinical cases in both humans and animals (Feder et al. 2012).

Despite rabies being eradicated from several developed countries, it is still a major zoonosis present in more than 150 countries, causing more than 59 000 human deaths each year; among them, 40% are children less than 12 years old (WOAH 2023). This disease affects all warm-blooded mammals, but the dog is the main reservoir of the virus in the African and Asian continents (WOAH 2023). In Tunisia, a similar trend is observed because 61% of infected animals are dogs (Brour 2021; Ripani et al. 2017).

Other animal species, such as captive primates, could be infected when they are exposed to rabid dogs (Bais, Tak & Mahla 2017), which presents both conservation and public health problems. Indeed, among travellers, primates are the second most common animal species, after dogs, to cause injuries requiring post-exposure prophylaxis for rabies (Gautret et al. 2014).

Zoos are the ideal environments where contact between humans and captive primates occurs (Esposito et al. 2023). Several primate species, such as baboons and lemurs, share spaces where interactions with visitors are frequent, increasing the risk of zoonoses (Bender & Shulman 2004; LeJeune & Davis 2004). The risk of rabies transmission from wild to domestic animals poses a real threat and was reported by several authors (Butler, Du Toit & Bingham 2004; Lembo et al. 2008; Mills & Hofer 1998; Wimalaratne & Kodikara 1999; Woodroffe & Donnelly 2011).

Thus, the vaccination of wild animals and especially primates against zoonoses plays an important role in increasing resistance of animals and prevents human transmission (Monath 2013). As captive non-human primates (NHP) are exposed to rabies in countries where the disease is endemic, it is crucial to protect them by vaccination. Human rabies vaccines are expensive and require multiple doses for the induction of protective immunity (Xiang et al. 2014). Moreover, in middle-income countries like Tunisia, where 12 076 post-exposure prophylaxis treatments were administered in 2021 (Pasteur Institute of Tunis 2021), the health ministry store rabies vaccines for human use only. Thus, only animal vaccines are available to protect non-human primates against rabies. However, these vaccines are not systematically tested in wild animal species and a gap of knowledge regarding their efficacy exists. Indeed, commercial, adjuvanted, inactivated rabies vaccines are authorised for use only in dogs, cats, cattle and horses, without specific mention for non-human primates (Martelli et al. 2022). As far as we know, management protocols for primates in captivity in zoos do not present clear and standardised data regarding rabies vaccination for this animal species.

As part of the One Health approach, it is of paramount importance to guarantee the safety of animals and humans and to protect them against rabies virus infection risk. The present study aims to examine the kinetics of rabies antibodies after vaccination of different groups of primates with an inactivated and adjuvanted rabies vaccine.

The outputs could serve as a reference point for a possible standardised protocol for the vaccination against rabies of primates in captivity and more broadly of warm-blooded mammals in the zoo.

## Research methods and design

### Study area

**FIGURE 1:**
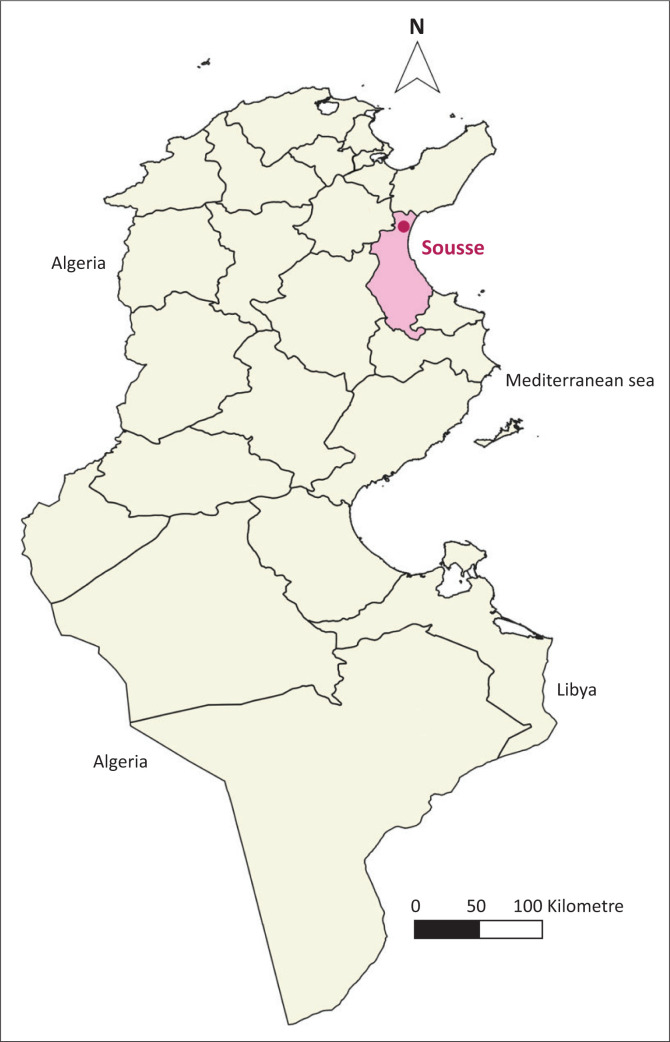
Map of Tunisia, showing the district of Sousse where the Friguia zoo Park is located (red dot).

Friguia zoo Park is located near the town of Bouficha (Sousse Governorate), 90 km from the capital, Tunis (Figure 1). The park opened its doors in November 2000 and is considered the largest park housing wild animal species in Tunisia.

This park spans an area of 36 ha and houses several animal species, such as tigers, lions, crocodiles, giraffes, elephants and wild small mammals. The primates are kept in cages at night and released during the day into enclosures ranging between 800 m^2^ to 1000 m^2^.

### Animals

The non-human primate population consists of 33 individuals belonging to the species of Guinea baboons (*Papio papio*), Barbary apes (*Macaca sylvanus*), black lemurs (*Eulemur macaco*) and ring-tailed lemurs (*Lemur catta*). The total number comprises 17 males and 16 females, with an average age of 11 years, range 2–21 years (Table 1). The primates are regularly monitored by a veterinarian and are considered to be in good health.

**TABLE 1:** Demographic characteristics of the non-human primates included in this study.

Name (family, species)	Number	Male: Female	Mean age (years)	Min–Max (years)	Mean weight (kg)	Min–Max (kg)
Guinea baboons (Cercopithecidae, *Papio papio*)	11	4:7	13.3	5–21	15.6	10.8–23.4
Barbary apes (Cercopithecidae, *Macaca sylvanus*)	9	3:6	Unknown	-	7.8	6–10
Black lemurs (Lemuridaes, *Eulemur macaco*)	6	5:1	Unknown	-	2	1.2–2.7
Ring-tailed lemurs (Lemuridaes, *Lemur catta*)	7	5:2	8	2–13	1.7	1.1–2.1

Min, minimum; Max, maximum.

### Protocol design

The experimental protocol involved a single primovaccination injection performed on 20 June 2022. Blood samples were collected at day one (D1), 1 month (D30), 6 months (D180) and 12 months (D365) post-vaccination. After anaesthesia, the primates were captured, microchipped, vaccinated and dewormed on the date of their initial rabies vaccination. Deworming procedure consisted of a single subcutaneous injection of ivermectin at the dose of 0.2 mg/kg (Ivermectyl, Medivet, Tunisia).

### Anaesthesia

For Barbary apes and Guinea baboons, an anaesthetic protocol is used to ensure the restraint and protection of both handlers and animals. Five mg/kg of ketamine (Ketamine^®^ 1000, Virbac, France) combined with 0.1 mg/kg of medetomidine (Domitor^®^, Orion Pharma, Finland) is prepared in a blowpipe-syringe and administered in the gluteal muscle using a blowpipe by the park’s veterinarian.

### Vaccination

The vaccine used for this study was Rabisin^®^ (Bohringer, Germany), the only animal rabies vaccine available on the Tunisian market at the time of the study. It is an inactivated virus vaccine, marketed in a 1 mL vial, containing the fixed Pasteur strain ‘G52’ (≥ 2.09 log_10_ DO_50_ and > 1 IU) and adjuvanted with aluminium hydroxide (1.7 mg/mL). This vaccine is authorised for use in Tunisia at a dose of 1 mL via the subcutaneous route for dogs, cats and cattle and via the intramuscular (IM) route for equids.

The vaccine was administered in the medial deltoid muscle at a dose of 1 mL. The IM route was preferred over the subcutaneous route because it is more convenient for administering the vaccine using a blowpipe, mainly for larger primates.

### Sampling

The animal was immobilised on an examination table in lateral decubitus position. A tourniquet was placed above the left arm pit, which was shaved and disinfected with 70% alcohol. A clinical examination was performed at the same time, and medical care was provided when the animals were injured.

Blood was collected from the superficial radial vein for Guinea baboons and Barbary apes and the femoral vein for black lemurs and ring-tailed lemurs. The blood collection (5 mL for baboons and apes and 2 mL for lemurs) was performed using a XINLE 21G1/2 needle attached to a dry vacuum tube (identified with the animal’s chip number). Once transported to the local laboratory, the samples were centrifuged for 15 min at 5000 rotation per minute (rpm). The obtained sera were transferred to Eppendorf microtubes with the same identifier and frozen until shipment to the national reference laboratory for rabies at the Pasteur Institute of Tunis. All samples were shipped in coolers with ice packs.

### Serology

The titration of rabies-neutralising antibodies was performed using the fluorescent antibody virus neutralisation (FAVN) test, as recommended by the World Organisation for Animal Health (WOAH 2023), at the Pasteur Institute of Tunis. Briefly, the serological test (FAVN) was carried out as outlined by Cliquet, Aubert and Sagné (1998). This test is based on serum neutralisation using the ‘challenge virus standard’ (CVS-11) strain and BHK-21 cells. The Fujirebio anti-Rabies Nucleocapsid Conjugate was employed for staining CVS-infected cells. To reduce internal variance, sera from each sampling time were titrated in the same series. The antibody titre refers to the dilution of serum at which 50% of the virus is neutralised in the test wells, indicating the level of neutralising rabies antibodies present. Observations of the wells under a fluorescent microscope enable the calculation of the dilutions required to neutralise 50% of the virus in logD_50_. To convert logD_50_ to IU/mL units, a computation is performed using the logD_50_ value of the series’ reference serum. In this study, we utilised the reference serum ‘Human rabies immunoglobulin BRP batch 1’ (91 IU/vial), diluted to 0.5 IU/mL, tested and validated through an interlaboratory test established by the European reference laboratory for rabies. The threshold is set at 0.5 IU/mL (WOAH 2023), and titres below this value indicate non-immunised animals.

### Statistical analyses

Data (age, sex, chip number, etc.) were initially collected using Kobo Toolbox® (Harvard Humanitarian Initiative, United States). The collected information was transferred to an Excel sheet (Microsoft, United States) and gradually completed with laboratory results of rabies neutralisation tests at each sampling date.

The comparisons of frequencies (proportion of primates yielding a titre different from the threshold) were conducted using a χ^2^ (Chi-squared) test or Fisher’s exact test for small samples at a significance level α ≤ 0.05 (Schwartz 1993).

To avoid skewness results and negative values, all the titres were log transformed as following: ln (titre IU/mL+1) and compared to the protective threshold value of 0.41 UI/mL (equivalent to 0.5 IU/mL).

Comparisons of the mean rabies-neutralising antibodies-transformed titres estimated at post-vaccination sampling dates (Day 30, Day 180, Day 365) with the mean pre-vaccination titre (D1) were carried out using a paired samples *t*-test (each subject is considered as its own control).

As NHP species number is low, comparisons of the mean rabies-neutralising antibodies-transformed titres, between species and sexes at each sampling date, were performed using Kruskal-Wallis test and Mann-Whitney *U*-test, respectively (Schwartz 1993).

A correlation test was conducted to examine the relationship between quantitative variables: age and weight of primates with the median transformed titres expressed throughout the year. The Spearman correlation coefficient (*r*) was calculated. The closer ‘*r*’ is to zero, the weaker the linear relationship. The value of ‘*r*’ is meaningful only if it is statistically significant at the 0.05 threshold (Schwartz 1993).

All statistical tests were considered significant at the threshold α ≤ 0.05.

Statistical analyses were conducted using the SPSS (Statistical Package for the Social Sciences) version 26 (IBM, United States).

### Ethical considerations

Ethical clearance to conduct this study was obtained from the National School of Veterinary Medicine of Sidi Thabet (Tunisia) (No. CEEA-ENMV 76/24).

## Results

### Evolution of the rabies-neutralising antibody titres among the captive non-human primates

In comparison to D1, the overall mean antibody-transformed titres evolved significantly at D30, D180 and D365 (*p* < 0.0001). Only one ape showed significant titres (≥ 0.41 IU/mL) prior to rabies vaccination. One-month post-vaccination, 100% of primates developed titres ≥ 0.41 (IU/mL) (Table 2). A variation of humoral responses was reported at D180 and D365 with 91% (*n* = 30/33) and 76.5% (*n* = 25/33) of primates, respectively maintained antibody titres above the threshold value (Table 2).

**TABLE 2:** Evolution of rabies-neutralising antibody-transformed titres parameters from D1 to D30, D180 and D365 post-vaccination.

Characteristics and parameters	D1	D30	D180	D365
Mean	0.08	6.4	4.02	1.3*
95% CI	0.04–0.1	5.6–7.2	2.9–5.1	0.8–1.8
Median	0.03	7.92	4.56	0.87
Range (minimum–maximum)	0.03–0.5	1.51–7.92	0.22–7.92	0.07–4.56
**Primates having titres ≥ Threshold ln(titre UI/mL+1)/Total number of primates examined)**				
%	3	100	91	76.5**
*n*	1	33	30	25
*N*	33	33	33	33
**Number of primates having titres ≥ Threshold ln(titre UI/mL+1)/Number of primates examined) according to sex**				
Males				
*n*	0	17	16	14
*N*	17^ns^	17^na^	17^ns^	17^ns^
Females				
*n*	1	16	14	11
*N*	16	16	16	16
**Number of primates having titres ≥ Threshold ln(titre UI/mL+1)/Number of primates examined) according to species**				
Baboons				
*n*	0	11	10	7
*N*	11^ns^	11^na^	11***	11^ns^
Barbary apes				
*n*	1	9	7	5
*N*	9	9	9	9
**Lemurs (black and ring-tailed)**				
*n*	0	13	13	13
*N*	13	13	13	13

^ns^, not significant using ‘Fisher’ test; ^na^, not applicable; D, day; CI, confidence interval.

* , Significant using ‘Student’ *t*-test for paired samples (D1 as a reference);

** , significant using ‘Chi-square’ test;

*** , significant using ‘Fisher’ test.

One-year post-vaccination, although a downward trend was observed for all primates, 76.5% (*n* = 25/33) maintained titres above the protective threshold value, and 24.2% (*n* = 8/33) animals had titres below the critical value, going as low as 0.07.

**FIGURE 2:**
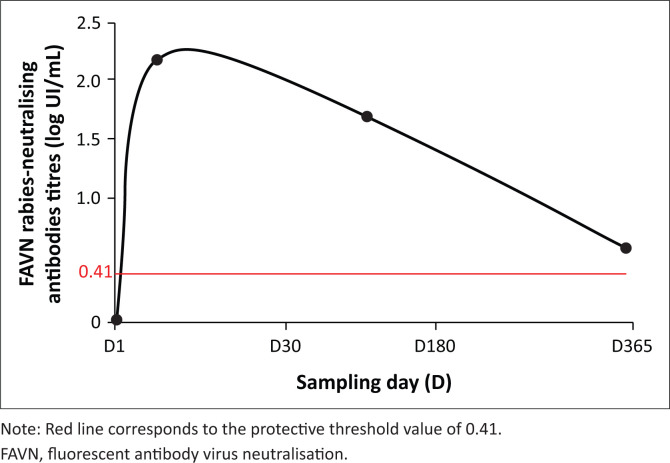
Overall median of rabies-neutralising antibody-transformedtitres in non-human primates prior to rabies vaccination (D1) and at D30, D180 and D365 post-vaccination.

The median antibody-transformed titres peaked 1 month (D30) post-vaccination and decreased slowly at D180 and D365, remaining above the threshold value (Figure 2).

### Evolution of the mean rabies-neutralising antibody titres according to the species and sex of the captive non-human primates

The mean rabies-neutralising antibody titres were significantly different between the four NHP species at each sampling date, excepting at D1 (Table 3). At D30, the lemurs displayed higher mean rabies-neutralising antibody titres than baboons (*p* = 0.002). Then, at D180, the lemurs displayed again higher mean rabies-neutralising antibody titres than baboons (*p* = 0.023 [black lemurs]; *p* = 0.039 [ring-tailed lemurs]) and Barbary apes (*p* < 0.001). And at D365, the lemurs displayed higher mean rabies-neutralising antibody titres than Barbary apes (*p* = 0.002) (Table 3).

**TABLE 3:** Significant transformed titres for pairs of non-human primates’ species using Kruskal-Wallis test for independent samples.

Sampling day	Mean transformed titre (UI/mL)	Median	Baboon	Barbary ape	Black lemurs	Ring-tailed lemurs	*p*-value*
**D1**	-	-	na	na	na	na	0.053
**D30**	-	-	-	-	-	-	0.005
Baboon	1.65	1.72	-	0.083	**0.002**	**0.004**	-
Barbary ape	1.93	2.19	-	-	0.188	0.169	-
Black lemurs	2.19	2.19	-	-	-	1	-
Ring-tailed lemurs	2.19	2.19	-	-	-	-	-
**D180**	-	-	-	-	-	-	< 0.001
Baboon	1.28	1.29	-	0.085	**0.023**	**0.039**	-
Barbary ape	0.6	0.63	-	-	**< 0.001**	**< 0.001**	-
Black lemurs	2.07	2.07	-	-	-	0.777	-
Ring-tailed lemurs	1.96	2.19	-	-	-	-	-
**D365**	-	-	-	-	-	-	0.002
Baboon	0.65	0.63	-	0.157	**0.044**	0.057	-
Barbary ape	0.32	0.41	-	-	**0.002**	**0.002**	-
Black lemurs	1.12	1.1	-	-	-	0.855	-
Ring-tailed lemurs	1.04	0.92	-	-	-	-	-

Note: Bold characters: significant values.

na, not applicable; D, day.

* , Summary of the Kruskal-Wallis statistic for independent samples, significant at α ≤ 0.05.

**FIGURE 3:**
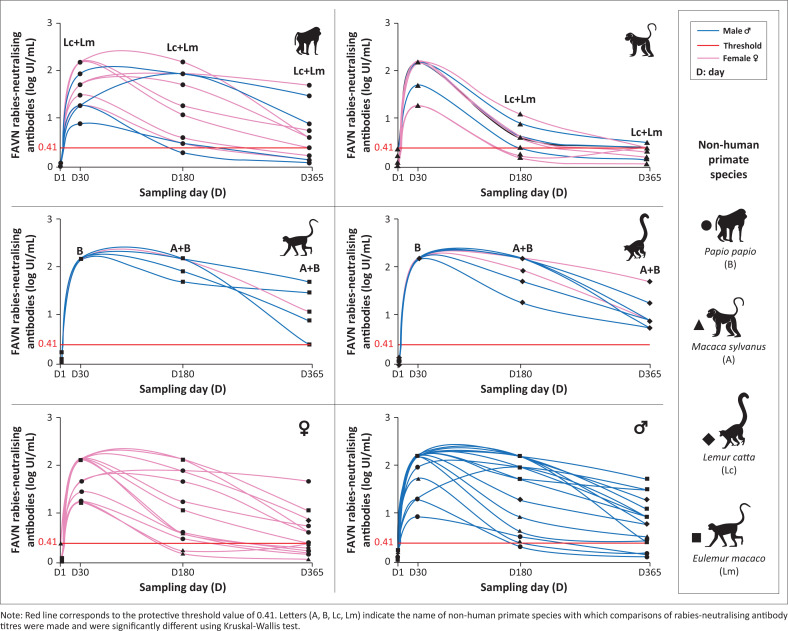
Mean rabies-neutralising antibody titres in non-human primates prior to vaccination (D1) and at D30, D180 and D365 post-vaccination according to species and sex.

At D365, all the lemurs maintained high antibody titres (Figure 3).

Using the *U*-test of Mann-Whitney, there were no significantly different means of rabies-neutralising antibody titres between males and females at each sampling date. Moreover, among the sampled females, 6 were pregnant during the study and gave birth as late as 19 July 2023.

No correlation was evidenced between the mean rabies-neutralising antibody titres and the age or weight of primates.

## Discussion

Rabies is enzootic in Tunisia and represents a public health threat. Although, in the Friguia zoo Park, the entry of dogs is not allowed, cats are abundant, attracted by animals’ food and restaurant trash. Moreover, multiple visitors abandon their sick cats in the park, hoping that the veterinarian takes care of them. Thus, vaccinating captive primates is of paramount importance for the protection of both the animals and the people who come into close contact with them.

Thirty-three captive non-human primates from four different species received an adjuvanted and inactivated dog vaccine through intramuscular injection. The kinetics of neutralising antibody was measured the first day (D1), the day 30th, the day 180th and the day 365th post-vaccination.

At D1, an antibody titre higher than the threshold (0.41) was detected in one ape. Several hypotheses can be advanced to explain this observation. The ape was previously confiscated by the Forestry Department (Ministry of Agriculture), and it may be possible that it was vaccinated against rabies after its capture. Indeed, isolated apes have been known to cross the north-western border of Tunisia from Algeria, where they live freely in forests and natural parks (Bergin, Atoussi & Waters 2018). To protect the country from primate-borne zoonotic viruses, the Tunisian government prohibits the importation and transit of all species of primates since 2014 (Journal Officiel de la République Tunisienne 2014). All captured primates are systematically transferred to Friguia zoo Park after receiving rabies vaccination.

The other hypothesis is that this animal may have been exposed to viral strains similar to Rabies virus (RABV) and expressed abortive form of rabies. Indeed, abortive rabies is caused by certain strains that engender disease in humans and animals without causing health anomalies (Benavides et al. 2020; Centers for Disease Control and Prevention [CDC] 2010; Feder et al. 2012), but this is still a hypothesis in the present study.

To evaluate the rabies antibody response post-vaccination in the non-human primates, using the FAVN method, we used the cut-off value of 0.5 IU/mL as recommended by the WHO and WOAH for humans and dogs and cats, respectively. Despite there is no validation of this cut-off in all the animal species, it is assumed that it could be used for non-human primates as a protective threshold (Gold et al. 2020; World Health Organization 2018; Xiang et al. 2014). However, further investigations are needed to assess the cellular immune response in non-human primates and provide insightful conclusion.

In our study, 100% of animals achieved protective titres (≥ 0.5 IU/mL) by 30 days post-vaccination, which is consistent with the results of Napolitano et al. (2020), where all vaccinated animals reached protective titres within 2 weeks, and Kessler, Summer and Baer (1982), where 8 out of 10 monkeys had titres above 0.5 IU/mL at the same date. Similarly, the long-term persistence of titres observed in our study – 76.5% of animals maintaining titres above 0.5 IU/mL at 1 year – aligns closely with the findings of Napolitano et al. (2020), where titres remained stable for up to a year.

However, differences emerge in the timing of peak titres, the rate of decline and the variability between species. In our study, peak titres occurred at 30 days, while Napolitano et al. (2020) reported peak titres between weeks 2 and 4. This discrepancy likely arises from differences in sampling intervals; titres at 2 weeks were not measured in our study. The vaccine formulation also plays a significant role, as Napolitano et al. (2020) used ChAd155-RG, a chimpanzee adenovirus-based vector designed for rapid and robust immune induction. Whereas in the present study, an adjuvanted and inactivated rabies vaccine (Rabisin^®^) was used. Similarly, Kessler et al. (1982) and Passos et al. (2001) used killed suckling mouse brain vaccines (SMBV), which demonstrated high titres after two doses and a significant decline in titres by 4 months, respectively.

Species-specific differences account also for variations, because Guinea baboons, Barbary apes, black lemurs and ring-tailed lemurs were included in the present study and showed different antibody kinetics. The comparison with other studies must be done carefully because different non-human primates were used: macaques (Lodmell et al. 1998; Napolitano et al. 2020), rhesus monkeys by Kessler et al. (1982) and capuchin monkeys by Passos et al. (2001). In our study, lemurs showed higher and sustained titres up to 1 year, while baboons and Barbary apes experienced a sharper decline. Such variability underscores the influence of species-specific immune dynamics on vaccine efficacy.

Lastly, the number and timing of vaccine doses and the route of administration also contribute to differences. Our study employed a single primovaccination, whereas Kessler et al. (1982), Passos et al. (2001) and Lodmell et al. (1998) included multiple doses or booster shots. Boosters enhance long-term antibody responses, as observed in Kessler’s and Lodmell’s studies. Additionally, delivery methods such as intramuscular injection (Kessler et al. 1982; Napolitano et al. 2020) versus intradermal desoxyribonucleic acid (DNA) vaccination (Lodmell et al. 1998) significantly affect immune kinetics. These findings emphasise the need for standardised protocols that consider species-specific responses, vaccine formulations and administration routes to optimise immunisation outcomes in non-human primates.

Three primates (one baboon and two apes) exhibited antibody titres below the protective threshold at D180. The baboon is a subordinate individual and was deeply injured at D1 by another baboon on his cheek. Indeed, the effect of stress can be significant on the immune response (Dhabhar 2014), and this could explain the low antibody titres compared to the other baboons. The observed global decline in antibody titres at D180 most likely started earlier, which could not be possible to detect in the present study because of the fixed intervals of sampling dates, because of budgetary constraints. This decline in antibody titres was observed after D30 by Lodmell et al. (1998), at D122 by Passos et al. (2001) and at D45 by Napolitano et al. (2020) probably because of different protocols, vaccines and NHP species. However, the continuous titres’ increase starting at D28 until D210 observed by Lavender (1973) could be attributed to the intense vaccination protocol of seven daily 1 mL doses of cell culture vaccine. Therefore, it would be interesting to assess the evolution of antibody titres with at least two primovaccination injections, especially in baboons and apes.

The efficacy of rabies vaccines varies within a given animal species, and it is influenced by factors such as vaccine type, administration method and health status. For instance, De Oliveira et al. (2000) reported no detectable rabies antibodies in cattle vaccinated with an inactivated virus vaccine by the 6th week, whereas Sihvonen, Kuhnen and Neuvonen (1994) found that 42% of cattle vaccinated with Rabisin^®^ maintained titres above the protective threshold. Similarly, Harvey et al. (2016) observed that 85% of horses retained protective titres 1 year after vaccination with Rabisin^®^, highlighting the effectiveness of the intramuscular route. In contrast, dogs showed more variable responses. Almeida et al. (1997) noted that 74.1% of dogs vaccinated with SMBV were no longer protected 1-year post-vaccination, with nutritional status cited as the major contributing factor. Handous et al. (2023) found that only 7% of dogs with initially low titres were protected at D365 using Nobivac^®^, suggesting that mass vaccination conditions may influence outcomes.

These findings collectively underline the importance of considering vaccine type, administration protocol and the health and nutritional status of the animals, as well as the context of vaccination campaigns.

The selection of the IM route for vaccine administration was preferred because this method allows for remote injection using a dart gun, which significantly reduces stress. Although Nieves et al. (1996) obtained very satisfactory results using the subcutaneous route, their observation period was limited to 60 days. Martelli et al. (2022) also utilised the subcutaneous (SC) route but did not assess the outcomes in relation to the protective threshold.

Knowlton, Roetto and Briggs (2001) observed that the IM route yields higher and more persistent rabies-neutralising antibody titres than the SC in coyotes. Previous studies on dogs, such as those conducted by Bunn (2007) and Coyne et al. (2001), have indicated that the IM route can be more effective than the SC in inducing the humoral response.

Lemurs, the lightest and smallest species in our study, developed a satisfactory humoral response, suggesting that individual weight is a determining factor for the humoral response. There is no specific research establishing a relationship between primate species and their responses to vaccines. However, Kennedy et al. (2007) and Berndtsson et al. (2011) demonstrated that medium- to large-sized dogs were more likely to have antibody titres below 0.5 IU/mL. In contrast, smaller dog breeds were more likely to reach the protective threshold. This may explain why lemurs maintained high rabies antibody titres 1-year post-vaccination, but further investigations are required.

Sex is a biological variable that influences the innate and acquired immune response in several animal species (Klein & Flanagan 2016) and affects the humoral response during rabies vaccination as it has been demonstrated in cats (Mansfield et al. 2004). Although the rabies-neutralising antibody titres mean between males and females was not supported statistically, 5 out of 8 individuals not protected at D365 were females, and three of them were in early gestation at D180. Further exploration is needed to understand the potential correlation between gestation and the humoral response. On the other hand, 3 males were not protected up to 1-year post-vaccination; the zoo veterinarian reported that they were dominated by the others. Competition for resources within animal groups can influence various aspects of health, including immune function, mainly because of the stress induced (Cohen et al. 1992; Gust et al. 1991; Tung et al. 2012). Moreover, these individuals are malnourished because of inequitable access to food.

The age factor was not directly correlated with the humoral response in the present study, probably because the information on the age of certain individuals was missing. In dogs and cats, over 5 years old and 14 years old, respectively, there is a higher risk of developing titres below the protective threshold after rabies vaccination (Berndtsson et al. 2011; Mansfield et al. 2004). This is probably because of immunosenescence, a well-known phenomenon in humans, which is the decline in immune defence with age and was also described in wild animals (Peters et al. 2019).

The humoral response in animals in response to vaccine can be influenced by internal factors such as physiological status, genetic and immunological system and by the individual environment, including diet and stress (Rashid, Rasheed & Akhtar 2009). In the present study, the limited number of individuals may explain the absence of significant correlations between weight, age and antibody titres. In fact, the low number of individuals, the variation in species, age, sex and lifestyle of the non-human primate species included in this study could affect dramatically the generalisability of the results beyond the zoo population, and further investigations are required to validate the observations, though they are in concordance with other studies’ results. A large sample size with similar species, age, sex and physiological status could provide more accurate observations.

On the other hand, the present study did not assess the cellular response induced by rabies vaccination, which plays a crucial role in rabies immunity. Indeed, T and B lymphocytes, as well as natural killer (NK) cells, have a major role in this cellular response (Overduin, Van Dongen & Visser 2019). However, no data are available in the literature regarding the correlation between long-term (serological) immunity and the cellular and humoral parameters, in non-human primates and considering them similar to humans, must be done carefully. Indeed, the study by Ya et al. (2024) showed that in 78 humans bitten by confirmed rabies-positive dogs receiving post-exposition prophylaxis and equine rabies immunoglobulins, 87% seroconverted for antibody (≥ 0.5 IU/mL) and produced significantly elevated interleukin-4 (IL-4) and interferon-gamma (IFN-γ)-secreting T-cells after 14 days and up to 1 year. If certain individuals with titre below the threshold of 0.5 IU/mL are likely to be protected, this is still to be identified in further studies.

## Conclusion and recommendations

Rabies vaccination in captive primates using 1 mL of an adjuvanted, inactivated vaccine (AIV) by the IM route demonstrates satisfactory results, with 76.5% of vaccinated individuals maintaining titres above 0.5 IU/mL at D365. However, a decline in titres observed at 6 months (D180) in some species, particularly baboons and apes, suggests that booster vaccinations may be beneficial to sustain protective immunity in these individuals. Similar findings in studies on other species, such as cattle and dogs, have indicated the necessity of boosters to maintain antibody levels (Albas et al. 2013; Handous et al. 2023; Sihvonen et al. 1994). Based on this and our data, we propose either a single injection of primovaccination followed by a booster 6 months later and annual boosters thereafter or two injections at a 1-month interval followed by annual boosters. Further studies are required to optimise the vaccine volume according to the weight of primates and to ensure adequate food intake among subordinate individuals, as this may impact the humoral response.
